# Body image and brace wear adherence: preliminary results from brAIST

**DOI:** 10.1186/1748-7161-9-S1-O78

**Published:** 2014-12-04

**Authors:** Traci Schwieger, Shelly Campo, Stuart Weinstein, William Clarke, Briana Woods-Jaeger, Kelli Steuber

**Affiliations:** 1 The University of Iowa, Iowa City, Iowa, USA

## Background

There is no consensus regarding the relationship between body image and brace wear adherence in adolescents with adolescent idiopathic scoliosis (AIS).

## Aim

The aim of this study is to explore body image and brace wear adherence.

## Methods

This study used data from 171 BrAIST subjects that were in the randomized and preference brace treatment groups at baseline and did not switch to observation at any time during the study. Brace wear adherence categories were based on dose-response range efficacy findings in the BrAIST study. The three groups were: least adherent (0-6), middle adherent (6.1-12.8) and most adherent (≥ 12.9), using brace monitor data measuring hours/day in brace. The Spinal Appearance Questionnaire (SAQ)(1) was used to measure body image scores and scores were compared with largest Cobb angle, BMI, and quality-of-life measures. Wilcoxon ranked sum tests were conducted to test body image differences between the least adherent and the most adherent brace wear groups at baseline and at the 6, 12, 18, and 24 month follow-up visits.

## Results

There were no differences between subjects in the least adherent group and the most adherent group at baseline (n=36 and 95, respectively) or at any follow-up visits regarding BMI, largest Cobb angle, quality-of-life, and body image.

## Conclusions

Findings from this study do not support previous research indicating that wearing a brace may have a negative impact on body image, which could ultimately lead to poor brace wear adherence. In fact, this study found no difference in body image between the least adherent and the most adherent brace wear groups at any follow-up visit up to 2 years. Further analysis is being conducted to assess relationships between body image, quality-of-life, and demographic and clinical variables within brace wear adherence groups.

